# Resection of Bar in the Management of Calcaneonavicular Coalition: A Systematic Review

**DOI:** 10.7759/cureus.39275

**Published:** 2023-05-20

**Authors:** Vipul Garg, Omer Nasim, Sanjay Kumar, Mohammad Noah Khan, Abdullah Durrani, Arsallan Karim

**Affiliations:** 1 Trauma and Orthopaedics, Wrexham Maelor Hospital, Wrexham, GBR; 2 Trauma and Orthopaedics, Poole General Hospital, Poole, GBR; 3 Trauma and Orthopaedics, Gloucestershire Royal Hospital, Gloucestershire, GBR; 4 Trauma and Orthopaedics, University Hospitals Dorset NHS Foundation, Poole, GBR

**Keywords:** systematic literature review, laparoscopic surgery, open excision, calcaneonavicular, tarsal coalition, surgical resection

## Abstract

The most commonly encountered type of tarsal coalition in symptomatic patients is the calcaneonavicular coalition. Non-surgical treatments are effective for most patients. However, if surgery is required, excision of the calcaneonavicular bar can be a successful option that preserves hindfoot mobility and function.

We conducted a systematic review of calcaneonavicular bar excision in accordance with the Preferred Reporting Items for Systematic Review and Meta-Analysis Protocols (PRISMA-P) checklist. To conduct the review, we conducted a thorough search of several databases, including PubMed, Cochrane, Excerpta Medica Database (EMBASE), Cumulative Index to Nursing and Allied Health Literature (CINAHL), Google Scholar, and bibliographies. We analyzed the chosen studies to collect information on patient demographics, clinical outcomes, surgical techniques, and potential complications.

We identified 11 studies that included 274 patients for a total of 394 feet. The average age of patients in these studies was 12.5 years, ranging from 8.2 to 19.4 years. Follow-up periods varied from 2.3 to 23 years, with an average duration of 5.9 years. Excision of the calcaneonavicular bar was performed at 380 feet, while fusion was performed at 14 feet. In 50.5% of the feet, the extensor digitorum brevis was used as an interposition material. Successful outcomes after bar excision were observed in 82.9% of cases (304 feet) and were described as satisfactory, improved, good, or excellent outcomes. In one study, the American Orthopaedic Foot and Ankle Society (AOFAS) score improved from 47.89 to 90.22 in 12 feet after bar excision. Recurrence was reported in 52 feet out of the 380 feet that underwent bar excision. Progression of arthritis in the ankle and subtalar joint was reported in 25 feet. Various complications were reported, including paraesthesia in the hindfoot (three feet), midfoot pain (three feet), hindfoot pain (two feet), mild wound infection (one foot), and swelling and stiffness (one foot).

Surgical excision of the calcaneonavicular bar has shown successful outcomes in most patients, regardless of the use of interposition material. These outcomes are associated with minimal and acceptable complications. However, since the studies conducted in the literature were single-center retrospective and prospective trials, a multicenter prospective study with patient-centered, validated outcomes would provide a better opportunity to support the evidence in favor of surgical excision of the calcaneonavicular bar. Overall, the use of various interposition materials is associated with reduced chances of recurrence compared to cases where no interposition material was used.

## Introduction and background

Tarsal coalition is a well-known condition that is associated with peroneal spastic flat feet. Its relationship with painful, rigid flat feet was first highlighted in the landmark paper of Slomman [1], and later Badgley [1] further emphasized this relationship. The overall incidence of the tarsal coalition in various literature is reported to be between 1%-2%, with the coalition between the calcaneum and navicular being the most common (50%-60%) [2, 3]. The prevailing theory regarding the cause of tarsal coalition is the failure of the mesenchymal anlage of the foot to form properly. This coalition, which occurs between two tarsal bones, can be bony, cartilaginous, or fibrous [4].

**Figure 1:**
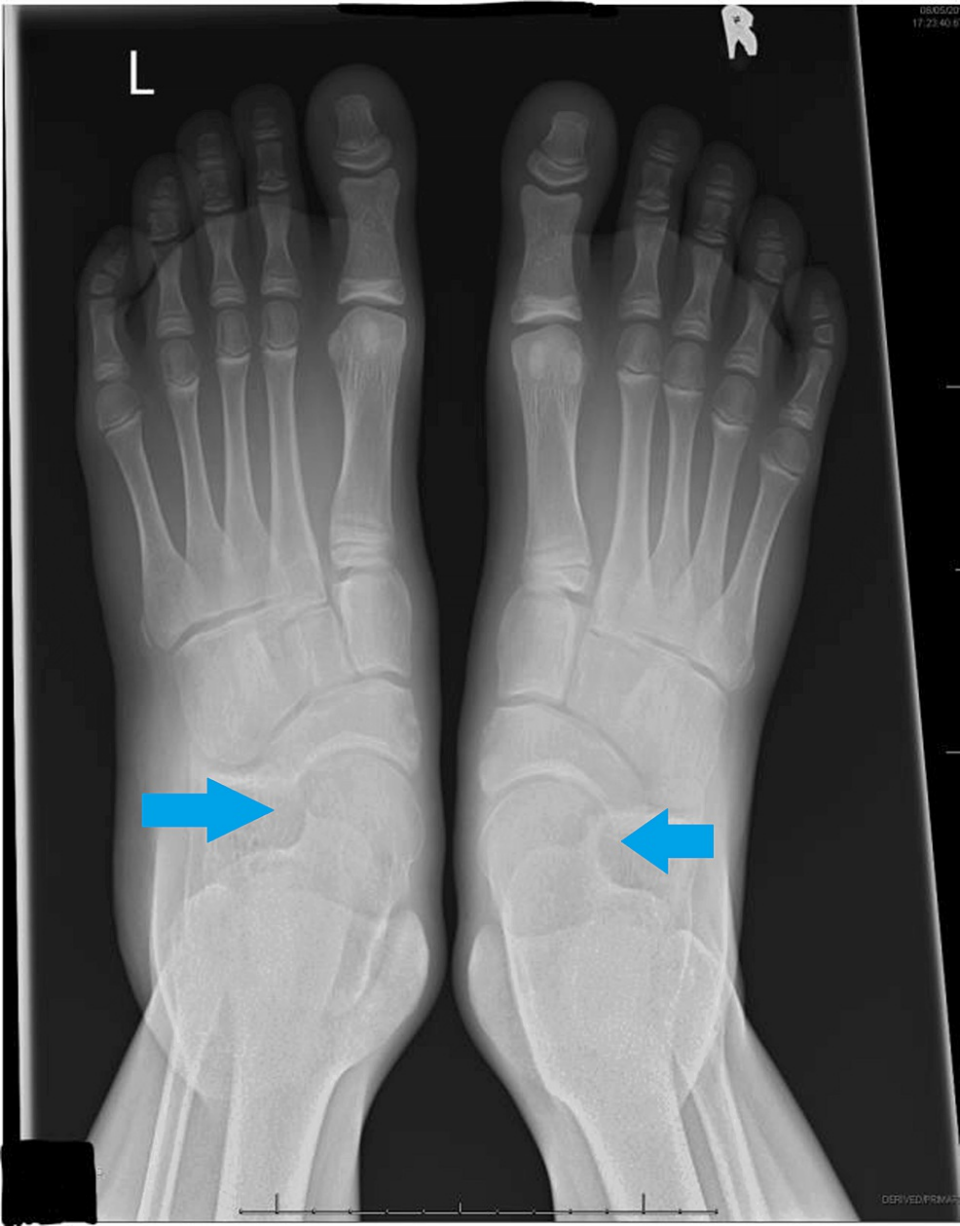
Radiograph of the bilateral calcaneonavicular coalition in the dorso pedal view. L: left; R: right

**Figure 2:**
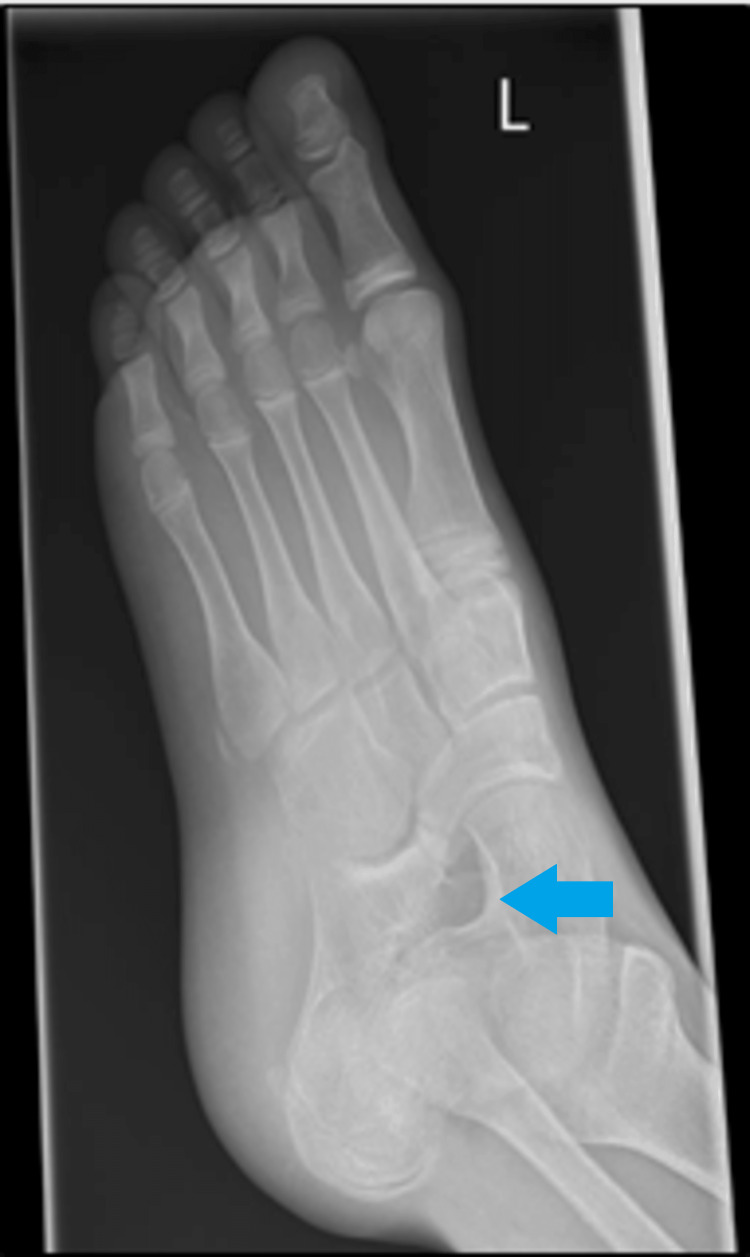
Oblique view of the foot with the bilateral calcaneonavicular bar. L: left

Calcaneonavicular coalition typically presents with persistent foot pain, repeated ankle sprains, and a rigid flat foot in children and adolescents aged eight to 14 years. The diagnosis is confirmed by using plain radiography, which reveals an extended anterior process of the calcaneum and a bar located between the calcaneum and navicular in oblique views. (as seen in Figures 1-2).

**Figure 3:**
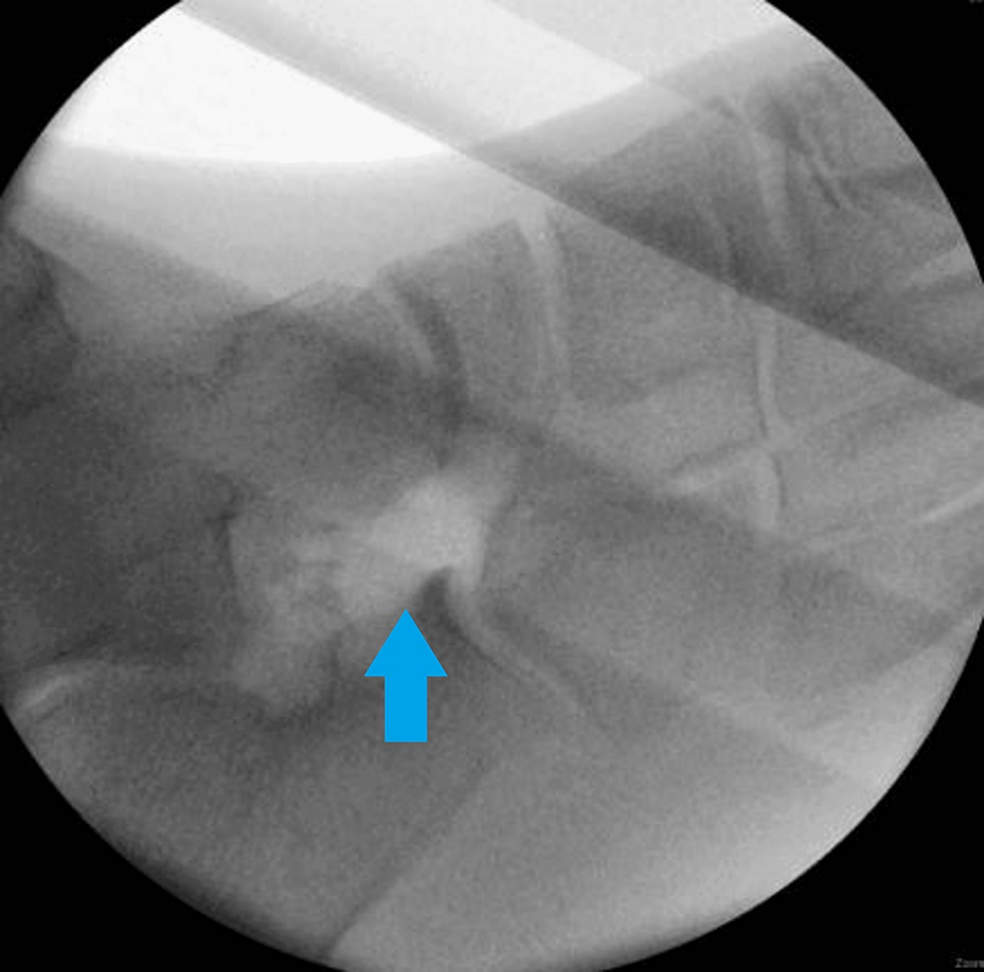
Surgical intraoperative photos showing excision of the calcaneonavicular bar.

**Figure 4:**
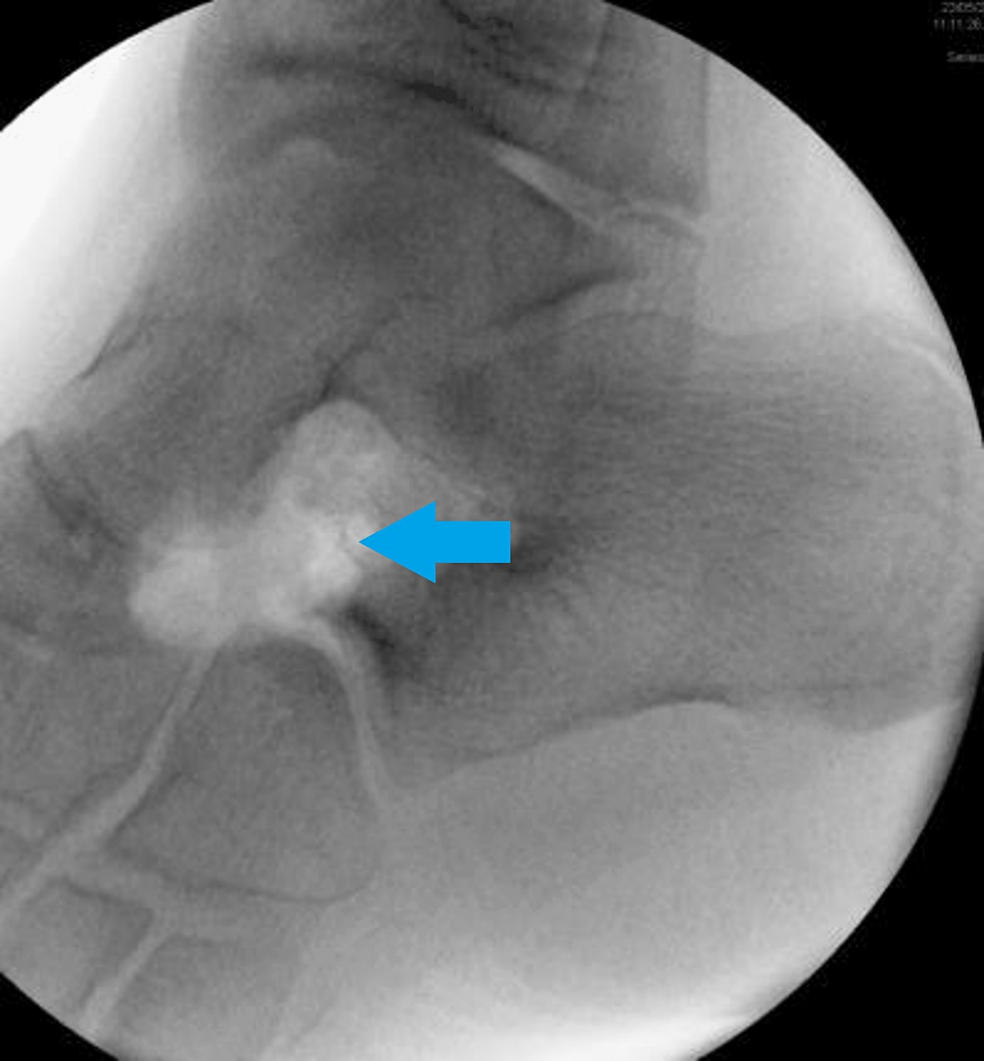
Surgical intraoperative photos showing excision of the calcaneonavicular bar.

Conservative treatment options such as taking nonsteroidal anti-inflammatory drugs, using medial arch supports, immobilization through a cast or foot or ankle orthosis, and local injection of corticosteroids in the subtalar joint should be attempted initially [5]. Only if these treatments are not effective, may surgical treatment be considered. Among surgical options, removal of the calcaneonavicular bar, with or without a natural or synthetic graft placed in between, is the preferred method. This can be seen in Figures 3-4.

Various studies have reported differing success rates in the surgical removal of calcaneonavicular bars, with or without the interposition of a graft. Our study aimed to conduct a systematic review of the surgical treatment of the calcaneonavicular coalition, specifically focusing on the clinical outcomes after resection. We aimed to gather and evaluate existing literature on the subject, as no systematic review has been conducted on this topic to date.

## Review

Protocol and registration

The systematic review was conducted following the Preferred Reporting Items for Systematic Reviews and Meta-Analyses (PRISMA) checklist [6]. The protocol was registered on Prospero (CRD42023411559).

Study search and inclusion criteria

The PRISMA-P 2015 checklist (Preferred Reporting Items for Systematic Review and Meta-Analysis Protocols) [6] was followed during the systematic review. PubMed, Cochrane, Excerpta Medica Database (EMBASE), Cumulative Index to Nursing and Allied Health Literature (CINAHL), Google Scholar, and bibliographies were searched to identify relevant research on outcomes in the tarsal coalition and calcaneonavicular coalition.

The search was built on combinations of the following terms: tarsal coalition, calcaneonavicular coalition, management, operative treatment, surgical resection, and outcome (as shown in Table 1). The dates were limited to January 1, 2006, until April 30, 2022. Additionally, references to full articles were reviewed and screened for missed publications. Exclusions were made based on language, with only English-language articles being included. Two independent reviewers conducted a systematic screening of references to determine their eligibility for inclusion in the study. In cases where relevant data were missing, the study authors were contacted to provide the required information, subject to available resources.

**Table 1:** Keywords searches for article identification.

Tarsal coalition AND calcaneonavicular coalition
Tarsal coalition AND management
Calcaneonavicular coalition AND management
Calcaneonavicular coalition AND operative treatment
Calcaneonavicular management AND surgical resection
Calcaneonavicular surgery and outcome

In this systematic review, the authors performed a comprehensive search of electronic databases using the specified keywords and conducted a screening process of the abstracts of relevant articles. The inclusion criteria consisted of randomized controlled trials, prospective or retrospective trials focusing on surgical treatment of the calcaneonavicular coalition through excision of the calcaneonavicular bar, and inclusion of patient demographic data, clinical outcomes, and complications. Inclusion criteria for this review involved randomized controlled trials (RCTs), quasi-RCTs, crossover trials, controlled before and after studies, interrupted time series studies, and prospective and retrospective cohort observational studies, both published and unpublished. Studies were required to have a minimum follow-up duration of 12 months, and only English-language studies meeting these criteria were considered for inclusion. Studies with predominant neurophysiological deformities and non-joint sparing procedures, such as arthrodesis, were excluded from the final analysis. Non-experimental studies were excluded due to the potential for a high risk of bias.

The data collection for this review involved recording patient demographic information such as the number of patients, age of patients, number of feet operated on, and duration of follow-up. Clinical outcomes were categorized as satisfactory or unsatisfactory, and the review also documented any associated procedures performed, postoperative management, reoperation, and complications.

Study selection

Inclusion criteria were assessed by examining titles or abstracts. Full-text articles were then read, and any that did not satisfy the inclusion criteria or met the exclusion criteria were excluded.

Observation and results

**Figure 5:**
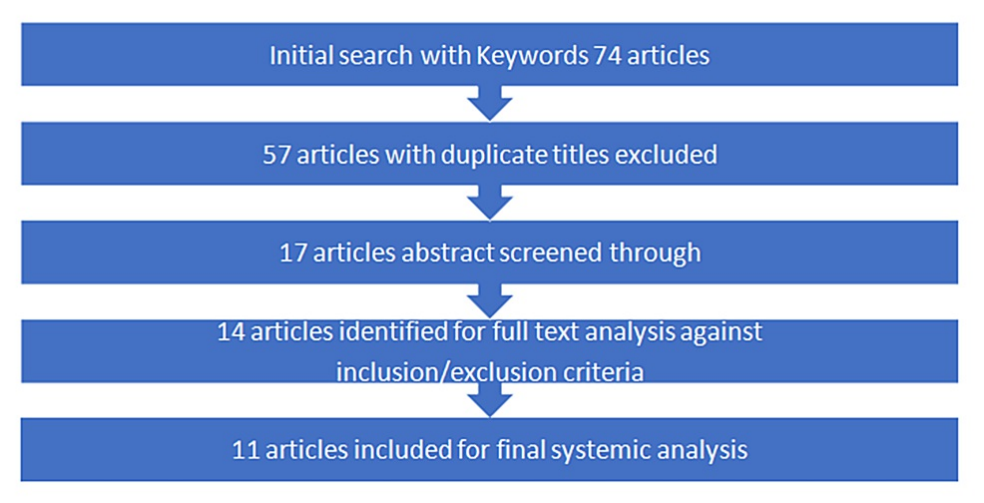
Flowchart of the article selection process for the systematic review on surgical management of calcaneonavicular coalition.

Upon conducting a search using the specified keywords, a total of 74 articles were initially identified. After removing duplicate titles, 57 articles were excluded. Out of the remaining 17 articles, duplicates were found in three of them, which were subsequently excluded. The remaining 14 articles underwent a full-text analysis, and after careful examination, 11 articles were deemed suitable for inclusion based on the established inclusion and exclusion criteria (Figure 5). These selected articles were then included in the systematic review. Their summarized version is presented in Table 2.

**Table 2:** A summary of procedures performed in various studies. EDB: extensor digitorum brevis

Study	Excision (No. of feet)	Arthrodesis (No. of feet)	Materials used for interposition
Andreason et al [3]	24	0	None
Moyes et al [7]	19	2	10: EDB, 3: None
El Shazly et al [8]	12	0	Synthetic graft
Alter et al [9]	17	0	EDB
Mubarak et al [10]	96	0	Fat
Cohen et al [11]	13	0	EDB
Gonzalez et al [12]	75	0	EDB
Mitchell et al [13]	42	0	None
Swiontkowski et al [14]	39	5	EDB
Chambers et al [15]	30	1	EDB
Ignis et al [16]	16	0	EDB
Total	354	8	

Demographics and follow-up

The systematic review included 11 studies that met the inclusion criteria, comprising a total of 274 patients who underwent surgical intervention for calcaneonavicular coalition, resulting in 394 operated feet. The mean age of the patients included in the studies was 12.7 years, with an age range of 8.2 to 19.4 years. Gender distribution was available for all studies except for two, with data from nine studies showing 169 males and 85 females. The mean follow-up duration reported in the 11 studies was 5.9 years, ranging from 2.1 to 23 years. The most frequently reported etiology in all studies was idiopathic. Data on clinical outcomes were unavailable for one patient due to a loss of follow-up. Table 3 displays the demographic distribution of the 11 studies.

**Table 3:** Demographic characteristics of patients included in the systematic review studies. (n) = Frequency; M: male; F: female

Serial No.	Studies included	Patients (n)	Feet (n)	Mean age (years)	Male/female ratio	Mean follow-up (years)
1.	Moyes et al [7]	14	19	12	-	3.4
2.	El Shazly et al [8]	9	12	12	-	2.3
3.	Alter et al [9]	14	16	19.5	M:8, F:6	4.5
4.	Mubarak et al [10]	69	96	12	M:47, F:22	2.5
5.	Cohen et al [11]	10	13	10.9	M:6, F:4	3.9
6.	Gonzalez et al [12]	48	75	11.2	M:32, F:16	23
7.	Mitchell et al [13]	28	42	11	M:18, F:10	6
8.	Swiontkowski et al [14]	30	44	12.2	M:21, F:9	4.6
9.	Ignis et al [16]	11	16	8.4	M:7, F:4	4
10.	Andreason et al [3]	22	25 (1 lost to follow-up)	9	M:13, F:12	10-22
11	Chambers et al [15]	19	31	20	M:17, F:2	8
Total		274	364	12.7		

Data analysis

**Figure 6:**
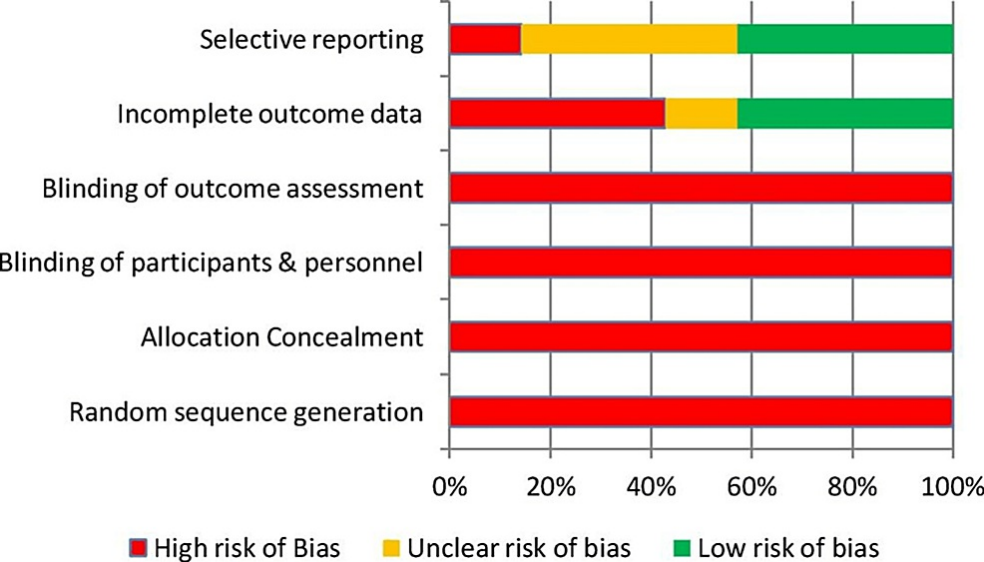
Cochrane risk of bias graph

Of the 11 studies included in this review, five were prospective and six were retrospective. None of the studies were randomized, which could lead to selection bias in data analysis. The inherent nature of prospective and retrospective studies also raises the risk of performance bias. The risk of bias assessment for this systematic review is presented in Table 4 and Figure 6.

**Table 4:** Authors' assessment of risk bias. (-) high risk of bias; (?) unclear risk of bias; (+) low risk of bias

	Sequence generation (random)	Concealment of allocation	Participants blinded	Outcome of blinding	Outcome data incomplete	Selective reporting
Moyes et al [7]	-	-	-	-	-	?
El Shazly et al [8]	-	-	-	-	-	-
Alter et al [9]	-	-	-	-	+	+
Mubarak et al [10]	-	-	-	-	?	-
Cohen et al [11]	-	-	-	-	+	+
Gonzalez et al [12]	-	-	-	-	+	+
Mitchell et al [13]	-	-	-	-	?	-
Swiontkowski et al [14]	-	-	-	-	+	?
Ignis et al [16]	-	-	-	-	-	-
Andreason et al [3]	-	-	-	-	+	-
Chambers et al [15]	-	-	-	-	+	?

Procedure

All the studies included in this systemic review utilized surgical excision of the calcaneonavicular bar as the primary treatment method for symptomatic calcaneonavicular coalition. In patients with radiological evidence of arthritis who were skeletally mature, primary arthrodesis was offered, resulting in seven patients (14 feet) undergoing primary arthrodesis of the calcaneonavicular joint, while 267 patients (380 feet) underwent excision. In the majority of the studies, interposition material was utilized following bar excision, with the extensor digitorum brevis (EDB) being the most frequently used interposition material in 175 feet (50.5%). Fat was used in 96 feet, synthetic materials such as Teflon and Dacron in 12 feet, and no material was used in 73 patients. In total, 322 feet (84.9%) were treated with interposition material along with bar excision. Open methods were employed for calcaneonavicular bar excision in all studies except one [17]. Further details on the procedures performed in each study can be found in the summarized procedures in Table 2.

Clinical outcomes

In most studies, the clinical outcome was evaluated based on criteria specified by the authors. Four studies used a measure of satisfactory or unsatisfactory clinical outcome, while three studies used measures of symptomatic improvement or no improvement. Two studies used measures of excellent, good, and poor outcomes. One study used the American Orthopaedic Foot & Ankle Society (AOFAS) scoring system, while another study used measures of subtalar motion, a single-limb functional test, and gait analysis. Table 5 summarizes the clinical outcomes reported in the included studies in this systematic review.

**Table 5:** Clinical outcomes included in the studies and their respective sample numbers. (n) = frequency

Study included	Satisfactory: (n) of feet	Unsatisfactory: (n) of feet
Alter et al [9]	12	4
Ignis et al [16]	11	5
Mubarak et al [10]	63	6
Mitchell et al [13]	31	10
Total	117	25
Study included	Improved: (n) of feet	No improvement: (n) of feet
Andreason et al [3]	22	2
Moyes et al [7]	13	4
Swiontkowski et al [14]	25	4
Total	70	10
Study included	Improved: (n) of feet	No improvement: (n) of feet
Cohen et al [11]	13	0
Gonzalez et al [12]	60	5
Total	73	5

Among the 364 feet that underwent calcaneonavicular bar excision, a successful outcome was observed in 304 feet (82.9%). Specifically, one study assessed the outcome using the AOFAS score and reported a mean score increase from 47.89 to 90.22 in 12 feet. In another study [15], various measures such as subtalar motion, single-limb functional tests, and gait analyses were employed to evaluate outcomes, revealing a significant improvement in 14 out of 18 feet that underwent bar excision.

Complications

Recurrence of calcaneonavicular bar after excision was reported in nine studies involving 52 feet, accounting for 13.6% of the total number of feet that underwent excision. Two studies reported arthritis of the ankle and subtalar joint at the final follow-up, involving 25 feet (6.7%). In eight studies, various complications were reported, including paraesthesia, subtalar pain, midfoot pain, wound infection, foot swelling, and stiffness. The overall incidence of complications other than recurrence and arthritis was reported as 3.1% (12 feet) in all eight studies. However, three studies reported no significant complications. Details of the complications reported in different studies are provided in Table 6.

**Table 6:** Complications associated with calcaneonavicular bar excision according to literature.

Paraesthesia	3
Subtalar pain	1
Midfoot pain	1
Mild wound infection	1
Swelling	1
Stiffness	1
Arthritis	2
Midfoot deformity	2
Recurrence	36

Surgical technique

Surgical resection is considered the preferred treatment modality for calcaneonavicular fusion, as proposed by Badgley [1]. This idea was later supported by Cowell [2], who suggested that the calcaneonavicular bar should be resected, especially in patients without any arthritic changes. Most authors have utilized an open approach to excise the bar.

The surgical technique for calcaneonavicular bar excision involves making a longitudinal incision below the sinus tarsi and dissecting through the extensor digitorum brevis (EDB) fascia to reach the fusion site. The proximal attachment of the EDB muscle is then elevated and reflected while preserving the superficial peroneal nerve dorsally and the long toe extensor and evertors. The fused bar is identified after lifting the EDB by its physis-like junction between the calcaneum and the navicular. A curette and rongeur are then used to remove the bar and reveal the underlying cartilage between the cuneonavicular joint. It is important to ensure the complete removal of the bar and all associated cartilage and bone from the calcaneus and navicular by excising a rectangle of bone (1x1cm). Care must be taken not to enter the talonavicular joint to prevent navicular displacement. The gap created after clearing the fused portion can be filled with various materials, including fat, EDB, or synthetic materials. Some authors have also used electrodiathermy to curate the cancellous surface to prevent regrowth, while others have not used any interposition material. The wound is closed in layers, and a short leg cast is typically applied for three weeks before starting mobilization. In one study, a bulky dressing was used for 48 hours postoperatively instead of a cast [8].

In summary, surgical resection of the calcaneonavicular bar is a commonly used approach, and the procedure involves careful identification and excision of the fusion bar. The gap established after excision can be filled with various materials, and the wound is closed in layers. A short leg cast is typically used postoperatively for three weeks, and early mobilization is initiated after its removal.

Discussion

Non-surgical interventions such as shoe inserts or casts have demonstrated promising outcomes for the treatment of calcaneonavicular coalition [18]. However, in cases where conservative treatments prove ineffective, surgical interventions, including resection, resection with interposition, and arthrodesis, are viable options [4]. Despite the availability of surgical techniques, there is a lack of systematic reviews in the literature that consolidate the findings of various studies on calcaneonavicular bar excision, which would be valuable for informed discussions with patients. To address this gap, a systematic review was conducted to gather and synthesize the results of multiple studies with moderate to long-term follow-up after surgery. The review encompasses patient demographics as well as clinical and radiological outcomes, providing clinicians with essential information to guide discussions with patients regarding the potential benefits, complications, and failures associated with calcaneonavicular bar excision.

Lemley et al. (2018) and Jayakumar et al. (2015) conducted extensive reviews on the current understanding of tarsal coalition and evaluated the available evidence for various treatments. Lemley's review proposed multiple treatment options based on the level and grade of evidence available in the literature. Both studies support the efficacy of calcaneonavicular bar excision in treating different types of tarsal coalitions, such as subtalar, talonavicular, and calcaneonavicular, in young patients without arthritic changes. The literature does not provide a consensus on the choice of interposition material following bar excision. However, the extensor digitorum brevis muscle (EDB) appears to be the most frequently used option in the majority of studies due to its ready availability and minimal complications from the donor site.

This systematic review showed that the most commonly used interposition material after calcaneonavicular bar excision was EDB (in 199 feet, or 50.5% of cases), followed by fat in 96 feet, and synthetic material in 12 feet. In 73 feet, no interposition material was used. Recurrence of the coalition was reported in 52 feet (13.6%) following bar resection. The use of EDB as the interposition material was associated with a lower rate of recurrence, with only three feet (<1%) affected. On the other hand, patients who received no interposition material had a higher rate of partial to complete recurrence, with 23 feet (32%) affected. One study by Moyes et al. [7] reported no recurrence in seven patients who underwent bar excision without any interposition material. Overall, the interposition of various materials appears to reduce the risk of recurrence compared to cases where no interposition material is used, as supported by previous studies [2,7,19].

Limitations

This systematic review has certain limitations that need to be considered. Firstly, the studies included in this review were all observational (retrospective and prospective) and conducted at a single center. Secondly, subjective criteria were used by most authors for outcome measurement, and various interposition materials were utilized, which made it challenging to isolate findings and make exact comparisons. Furthermore, these studies were susceptible to biases related to selection, allocation, and randomization because there was a lack of a control group. Additionally, due to the awareness of the operative intervention by the patients, surgeons, and data collectors, there was a risk of performance and detection bias.

Despite these limitations, we analyzed these studies for the completeness of patient information on the type of procedure, clinical outcome, and complications. It was observed that in adolescents with idiopathic etiology without arthritic changes, excision of the calcaneonavicular bar is a viable treatment option. In terms of timing, most studies performed excision on symptomatic patients who were skeletally mature. However, in the studies conducted by Alter et al. [9] and Chamber et al. [15], excision was performed in patients with an average age of 20 years, and satisfactory outcomes were achieved. It was noted that patients with arthritis had poor outcomes with excision, and fusion was the preferred method for dealing with recurrences in all studies. However, there was variability in outcomes among different studies regarding performing arthrodesis. As a result, a definitive conclusion regarding the appropriate indication and timing for these procedures is difficult to draw.

Two studies [8, 15] utilized objective assessment tools such as the AOFAS score and a functional test scoring system based on single-limb jumping, single-limb hopping, and single-limb standing to evaluate the outcomes of patients who underwent calcaneonavicular bar excision. These studies found that patients experienced improvements in their preoperative scores and reported little or no limitation in their desired lifestyle.

However, despite the favorable outcomes, recurrence and various complications were still reported following the excision of the calcaneonavicular bar. While three studies did not report any recurrence or complications, Andreason et al. (16/24) reported the highest incidence of recurrence, with an overall rate of 10.2% across all studies [3]. Furthermore, Gonzalez and colleagues [12] documented two instances of arthrosis that developed after calcaneonavicular bar excision and required later fusion. In eight studies, various complications were reported, such as paresthesia at the hindfoot and scar site (three feet), subtalar pain (three feet), midfoot pain (three feet), wound infection (one foot), foot swelling (one foot), and stiffness (one foot). Overall, the complication rate following calcaneonavicular bar excision was 3.1% (12 feet), which is considered an acceptable rate when compared to other surgical interventions performed for the peroneal spastic foot in the midfoot and hindfoot regions.

The literature suggests that while calcaneonavicular bar excision can result in positive outcomes, recurrence and complications still occur. It is essential to carefully consider the benefits and risks of the procedure in the context of the patient's individual needs and circumstances.

## Conclusions

Based on a systematic review of the literature, the excision of the calcaneonavicular bar, with or without interposition material, has demonstrated a satisfactory short- to medium-term outcome in 82.9% of patients. The recurrence rate following the excision is approximately 13.2%, while the rates of ankle or subtalar arthritis stand at 6.7%. In certain cases, primary arthrodesis has been performed as the initial treatment, resulting in no recurrence and mid-term evidence of arthritis in 14 feet. Complications, including paraesthesia, subtalar pain, midfoot pain, wound infection, foot swelling, and stiffness, have been reported at a rate of 3.1%.

However, the current body of evidence is insufficient to develop specific guidelines regarding etiology, associated procedures, interposition material selection, and post-operative protocols. To establish the efficacy of calcaneonavicular bar excision more definitively, high-quality multicenter randomized trials with long-term follow-up and the utilization of validated outcome scores that focus on patient experiences are warranted. Through such research endeavors, more robust recommendations and protocols for calcaneonavicular bar excision can be developed, applicable to diverse patient populations.
